# O-47 - INVESTIGATION INTO A POSSIBLE ENVIRONMENTAL SOURCE OF STEC O26 AT A FESTIVAL IN WALES, AUGUST 2025

**DOI:** 10.1093/pubmed/fdag046.042

**Published:** 2026-07-09

**Authors:** Mr Darren Beynon, Mrs Avril O'Gorman, Mr Albert Yung

**Affiliations:** Public Health Wales; Public Health Wales; Public Health Wales

## Abstract

**Background:**

In August 2025, an outbreak of Shiga toxin-producing *Escherichia coli* (STEC) O26:H11 linked to a family music festival in Ceredigion, West Wales was investigated. Initial laboratory notifications identified several genomically linked cases across Wales and Scotland. Given the location on agricultural land previously used for livestock grazing, concerns emerged regarding potential environmental exposure and wider transmission among attendees.

**Objectives:**

Identify the outbreak source, investigate epidemiological and genomic links, assess environmental and food related-risks, implement control measures to prevent further STEC transmission, provide guidance to affected residents and local partners, and identify public health recommendations for prevention and management of outbreak related to mass events.

**Methods:**

An Outbreak Control Team (OCT) undertook epidemiological, microbiological, and environmental investigations. Whole Genome Sequencing (WGS) was used to confirm case relatedness. Among 2,770 attendees, an analytical cohort study was conducted using an online questionnaire distributed to 1,057 Welsh-resident lead ticket purchasers, capturing information on symptoms, food/environmental exposures, hand hygiene practices, and behaviours. Risk ratios and multivariable regression were used to identify exposures associated with illness.

**Results:**

Five genomically identical, confirmed STEC O26 cases were identified across Wales and Scotland. The analytical study received 580 responses, identifying 39 probable/possible cases. Staying overnight at the festival was strongly associated with illness, as was reporting inadequate handwashing facilities. Consumption of chicken noodles appeared associated on analysis but was not significant after adjustment. Environmental investigations found the site had been used for cattle and sheep grazing 30 days before the event, consistent with a plausible environmental source.

**Conclusions:**

Evidence suggests an environmentally mediated outbreak, likely linked to residual livestock faecal contamination on fields. The investigation highlights the need to reassess current guidance on rest periods for grazing land prior to public events, improve early detection of non-O157 STEC, and enhance hygiene infrastructure at outdoor festivals to mitigate risk.

